# Bioimaging Tools Based on Polyelectrolyte Microcapsules Encoded with Fluorescent Semiconductor Nanoparticles: Design and Characterization of the Fluorescent Properties

**DOI:** 10.1186/s11671-019-2859-4

**Published:** 2019-01-18

**Authors:** Galina Nifontova, Anton Efimov, Olga Agapova, Igor Agapov, Igor Nabiev, Alyona Sukhanova

**Affiliations:** 1 Laboratory of Nano-Bioengineering, National Research Nuclear University MEPhI (Moscow Engineering Physics Institute), 31 Kashirskoye Shosse, Moscow, Russian Federation 115409; 2 V.I. Shumakov National Medical Research Center of Transplantology and Artificial Organs, 1 Schukinskaya str, Moscow, Russian Federation 123182; 3 Laboratoire de Recherche en Nanosciences (LRN-EA4682), Université de Reims Champagne-Ardenne, 51 rue Cognacq Jay, 51100 Reims, France

**Keywords:** Polymer microcapsules, Polyelectrolytes, Quantum dots, Bioimaging

## Abstract

Fluorescent imaging is a widely used technique for detecting and monitoring the distribution, interaction, and transformation processes at molecular, cellular, and tissue level in modern diagnostic and other biomedical applications. Unique photophysical properties of fluorescent semiconductor nanocrystals “quantum dots” (QDs) make them advanced fluorophores for fluorescent labeling of biomolecules or optical encoding of microparticles to be used as bioimaging and theranostic agents in targeted delivery, visualization, diagnostics, and imaging. This paper reports on the results of development of an improved approach to the optical encoding of polyelectrolyte microcapsules with stable, covered with the multifunctional polyethyleneglycol derivatives water-soluble QDs, as well as characterization of the optical properties, morphological and structural properties of the encoded microcapsules. The embedding of QDs into the polymer microcapsule membrane through layer-by-layer deposition on a preliminarily formed polymeric polyelectrolyte shell makes it possible to obtain bright fluorescent particles with an adapted charge and size distribution that are distinctly discernible by flow cytometry as individual homogeneous populations. The fluorescent microcapsules developed can be used in further designing bioimaging and theranostic agents sensitive to various external stimuli along with photoexcitation.

## Introduction

Development of fluorescent polymer micro- and nanoparticles to be used as carriers for targeted delivery of drugs, protein, and nucleic acid molecules is of special interest in the field of bioimaging and theranostic agents design [1–3]. Quantum dots (QDs) are 2- to 10-nm semiconductor colloidal crystals with the fluorescence peak wavelength depending on their physical size. A wide absorption spectrum and a narrow, symmetrical fluorescence spectrum with its position depending on nanoparticle size allow a single radiation source to be used for exciting fluorescence in a set of QDs with different fluorescence bands, which can be used for multiplexed detection. Therefore, QDs are very attractive and promising advanced fluorophores for diagnostics and imaging [4].

The use of polyelectrolyte microcapsules as carriers of various functional components makes it possible to develop a system responding to various physical (ultrasound, magnetic field, laser, or optical radiation) or chemical (pH, ionic strength of the microenvironment, and polarity of solvents) stimuli [5, 6]. The polyelectrolyte microcapsules are obtained using layer-by-layer deposition of oppositely charged polymeric polyelectrolytes onto a spherical matrix. The subsequent dissolution of the matrix yields a hollow structure whose stable polymer membrane consists of an interpolymer complex of polyelectrolytes [7–9]. The technique of layer-by-layer adsorption of polyelectrolytes allows various functional components, including magnetic, metal (gold or silver), or fluorescent (e.g., QDs) nanoparticles to be incorporated into the polymer membrane and the thickness of the membrane to be controlled as it is formed [10, 11].

Fluorescently labeled microcapsules are promising bioimaging agents that can be used to monitor their in vitro and in vivo transport and delivery [12, 13]. In the available methods of fluorescent labeling (optical encoding) of microcapsules, polymers are conjugated or physically mixed with fluorescent labels [14, 15]. The fluorescent components determining the optical properties of the microcapsules can also be incorporated inside them via co-precipitation of polymers labeled with fluorescent dyes during preparation of the matrix microparticles, e.g., calcium carbonate microspherolites [16]. They can also be encapsulated after the matrix is removed; for this purpose, diffusion of low and high molecular weight compounds through the polymer membrane is ensured by increasing the ionic strength or pH of the microenvironment. However, optical encoding of polyelectrolyte microcapsules with fluorescent nanocrystals is more promising due to their unique optical properties and effectiveness in bioimaging [17].

The known methods of encoding by incorporating QDs into the polymer membrane of polyelectrolyte microcapsules employ QDs water-solubilized with a low molecular weight ligand, e.g., thioglycolic acid or cysteine [18, 19]. The goal of this study was to develop highly stable fluorescent polyelectrolyte microcapsules optically encoded with water-soluble CdSe/ZnS (core/shell) QDs whose surface was additionally modified with a thiol derivative of polyethylene glycol (PEG) containing a carboxyl terminal group and to estimate the fluorescence and structure characteristics of the resultant fluorescent microcapsules.

## Methods

### The Aim, Design, and Setting of the Study

#### Fabrication of QD-Encoded Polyelectrolyte Microcapsules

CdSe/ZnS (core/shell) QDs with a fluorescence maximum at 590 nm coated with trioctylphosphine oxide (TOPO) were synthesized by Dr. P. Samokhvalov in the Laboratory of Nano-Bioengineering of NRNU MEPhI (Moscow, Russia). The QD purification and solubilization were carried as described earlier [20, 21]. TOPO was removed from the QD surface by dissolving the QDs in chloroform and subsequently precipitating them with methanol; the procedure was repeated three times. After that, the QDs were dissolved in chloroform again and precipitated with a cysteine solution in methanol at a QD-to-cysteine mass ratio of 1:0.13. The QD precipitate was washed of excess cysteine with methanol and dried in a vacuum concentrator. The dried QDs were resuspended in water with addition of 0.1 M sodium hydroxide. Afterwards, the dispersion was sonicated using ultrasound bath and filtered (pore size, 0.22 μm). To the resultant dispersion, a thiol derivative of PEG containing a terminal carboxyl group was added at a mass ratio of 1:4.6. The mixture was incubated overnight at 4 °C and PEGylated QDs were purified using gel filtration chromatography*.* The QD content of the samples obtained was determined spectrophotometrically at the wavelength of the first exciton. Solubilized QDs were characterized by hydrodynamic diameter and ζ-potential using dynamic light scattering and laser Doppler micro-electrophoresis by means of Zetasizer Nano ZS (Malvern, UK).

The QD encoding was performed using a modified technique of layer-by-layer deposition of oppositely charged polycation and polyanion polymers, as well as water-soluble QDs functionalized with carboxylated thiol derivatives of PEG, onto the surface of calcium carbonate microparticles obtained as described earlier [22]. The polymeric polyelectrolyte layers were formed out of the polycation poly(allylamine hydrochloride) (PAH) and the polyanion poly(sodium 4-styrenesulfonate) (PSS) or polyacrylic acid (PAA); the fluorophores were water-soluble PEGylated CdSe/ZnS QDs with a fluorescence peak at a wavelength of 590 nm, a ζ-potential of -26.7 ± 0.8 mV, and a hydrodynamic diameter from 18.7 to 23.3 nm. During the QD-encoded microcapsule, manufacturing process after each layer deposition the microparticle surface charge (ζ-potential) was controlled using laser Doppler micro-electrophoresis.

The calcium carbonate microparticles were resuspended in ultrapure water, and 0.5 mL of a 2 mg/mL PAH solution in 0.5 M NaCl was added. The suspension was sonicated in an ultrasound bath and incubated for 20 min while stirring at room temperature. After that, the excess polymer was washed off by centrifugation followed by resuspension in MilliQ water. For applying the next layer, consisting of the polymeric polyanion, the microbeads were resuspended in 0.5 mL of ultrapure water, and the suspension was mixed with 0.5 mL of a 2 mg/mL PSS solution in 0.5 M NaCl, sonicated in an ultrasound bath for 60 s, incubated for 20 min while stirring at room temperature, and washed of the excess polymer as described above. The washing of the microparticles after each stage of polyelectrolyte application was repeated three times. Before the encoding, five polyelectrolyte layers were applied onto the calcium carbonate microparticles, the fifth layer consisting of the polycation. After that, solubilized QDs were added, and the mixture was incubated while permanently stirring for 80 min. Then, six successive layers of oppositely charged polymers were applied, the sixth one consisting of polyanion PSS or PAA. Hollow polyelectrolyte microcapsules encoded with QDs were obtained by dissolving the calcium carbonate cores of the resultant shelled microbeads by washing them with 0.2 M disodium ethylenediaminetetraacetate (EDTA) (pH 6.5). After that, the microcapsule surface was additionally modified with bovine serum albumin (BSA) (Sigma-Aldrich, USA) by dispersing the microparticles in a 50 mM phosphate buffer solution (pH 7.4) containing 1% of BSA and subsequently incubating at 4 °C for 12 h in the dark. Shortly before use, the suspension of hollow microcapsules was washed of excess BSA five times with a 50 mM phosphate buffer solution (pH 7.4). The obtained polyelectrolyte microcapsules were stored at 4 °C in the dark.

#### Optical and Fluorescence Microscopies of the QD-Encoded Polyelectrolyte Microcapsules

The morphology and size distribution of the microparticles were analyzed using optical and fluorescence microscopies. In order to estimate the size distribution of the microparticles, we fixed 5 μL of the microparticle suspension in 10 μL of 50% glycerol on a slide. The samples were examined by means of an Axio Observer 3 microscope (Carl Zeiss, Germany) with an LD A-Plan 40x/0.55 M27 lens in a light field. Fluorescent images were obtained using an HBO 100 mercury illuminator (Burner Mercury) with an XF115-2 FITC longpass filter set, including a 505DRLP dichroic filter, a 475AF40 excitation filter, and a 510ALP emission filter (Omega Optical, USA), an EC Plan-Neofluar 100x/1.30 Oil Iris M27 lens (WD = 0.20 mm), a numerical aperture adjustable from 0.7 to 1.3, and Immersol 518F immersion oil (Carl Zeiss, Germany).

The morphological characteristics of the obtained microcapsules were studied in sections of the optically encoded polyelectrolyte microcapsules with a BSA-free surface fixed in epoxy embedding medium. For this purpose, the microcapsule suspension was sequentially dehydrated with 30, 50, 70, and 95% aqueous ethanol solutions and then treated with absolute ethanol (Acros Organics, USA) three times to ensure complete dehydration. Each stage of dehydration lasted for 15 min. Dehydrated samples of microcapsules were transferred to a 1:1 epoxy–ethanol mixture for 12 h and then to a 3:1 epoxy–ethanol mixture for 3 h. Then, the samples were transferred to a clean embedding epoxy media, and epoxy blocks were polymerized at 45 °C for 12 h and at 60 °C for 72 h. Then, 150-nm sections were cut from these blocks containing fluorescent QD-encoded polyelectrolyte microcapsules by means of a Leica EM UC6 ultramicrotome (Leica Microsystems, Austria) equipped with an Ultra AFM 35 diamond knife (Diatome, Switzerland) 2.0 mm in width. The sections were transferred onto a slide and examined under an Axio Vert.A1 fluorescent microscope (Carl Zeiss, Germany) using an HBO 100 mercury illuminator (Burner Mercury) for excitation and a 45 HQ TexasRed fluorescent filter set (*d* = 25 shift free (E), a 560/40 excitation BP, a FT 585 beam splitter, and a 630/75 emission BP) (Carl Zeiss, Germany) for recording fluorescence. Fluorescent images were obtained using a Carl Zeiss EC Plan-Neofluar100x/1.30 Oil Ph3 lens and Immersol 518F immersion oil (Carl Zeiss, Germany). The images were analyzed and processed using the Zen (Carl Zeiss, Germany) and Image J 1.48 v (USA) software.

#### Fluorescence Characteristics of the QD-Encoded Microcapsules

The fluorescence characteristics of the original QDs used for encoding and the QD-encoded microcapsules were analyzed using an Infinite 200 PRO multimodal plate reader (TECAN, Switzerland). Before the measurements, the plate with wells containing suspensions of QD-encoded microcapsules and microspheres was centrifuged using a 5810 R centrifuge (Eppendorf, USA) with an А-2-DWP rotor at 2630×*g* for 20 min. The fluorescence maxima of free QDs and QDs embedded in the polymer shell of the polyelectrolyte microcapsules were determined at an excitation wavelength of 480 nm; bottom-scanning mode was used for analysis of the samples.

#### Flow Cytometry

An FACSCanto II flow cytometer (Becton Dickinson, USA) equipped with a blue (488 nm) argon laser as an excitation source was used to analyze the samples of the original calcium carbonate microparticles, the microparticles with the QD-containing polyelectrolyte shell, and the QD-encoded hollow microcapsules. We analyzed 0.5-mL aliquots of a suspension containing 10^6^ microbeads/microcapsules; the number of collected events was 2500. The fluorescence intensity was recorded in the standard forward-scattered light (FSC), side-scattered light (SSC), and phycoerythrin (PE, 585/42 nm) channels. The data were processed using the FACSDiva software (Becton Dickinson, USA).

### Materials

We used carboxylated thiol derivatives of PEG containing a 12-monomer PEG spacer (Thermo Fisher Scientific, USA), poly(allylamine hydrochloride) (PAH) with Mw ≈ 15,000 Da (Sigma-Aldrich, Japan), poly(sodium 4-styrenesulfonate) (PSS) with Mw ≈ 70,000 Da (Sigma-Aldrich, USA), and polyacrylic acid (PAA) with Mw ≈ 15,000 Da (Sigma-Aldrich, USA). Sodium carbonate, calcium chloride, ethylenediaminetetraacetic acid disodium salt dehydrate, bovine serum albumin (BSA), epoxy embedding medium, and other reagents were from Sigma-Aldrich (USA). All working solutions were prepared using MilliQ water (18.2 mΩ cm) obtained by means of a Direct-Q water purification system (Millipore, France) and filtered through filters with a pore size of 0.22 μm.

### Statistical Analysis

The MS Office Excel 2007 and Origin Pro 2015 software packages were used for statistical analysis of the data. The results are presented as the means and standard deviations for three independent experiments.

## Results and Discussion

### Development of QD-Encoded Polyelectrolyte Microcapsules

**Fig. 1:**
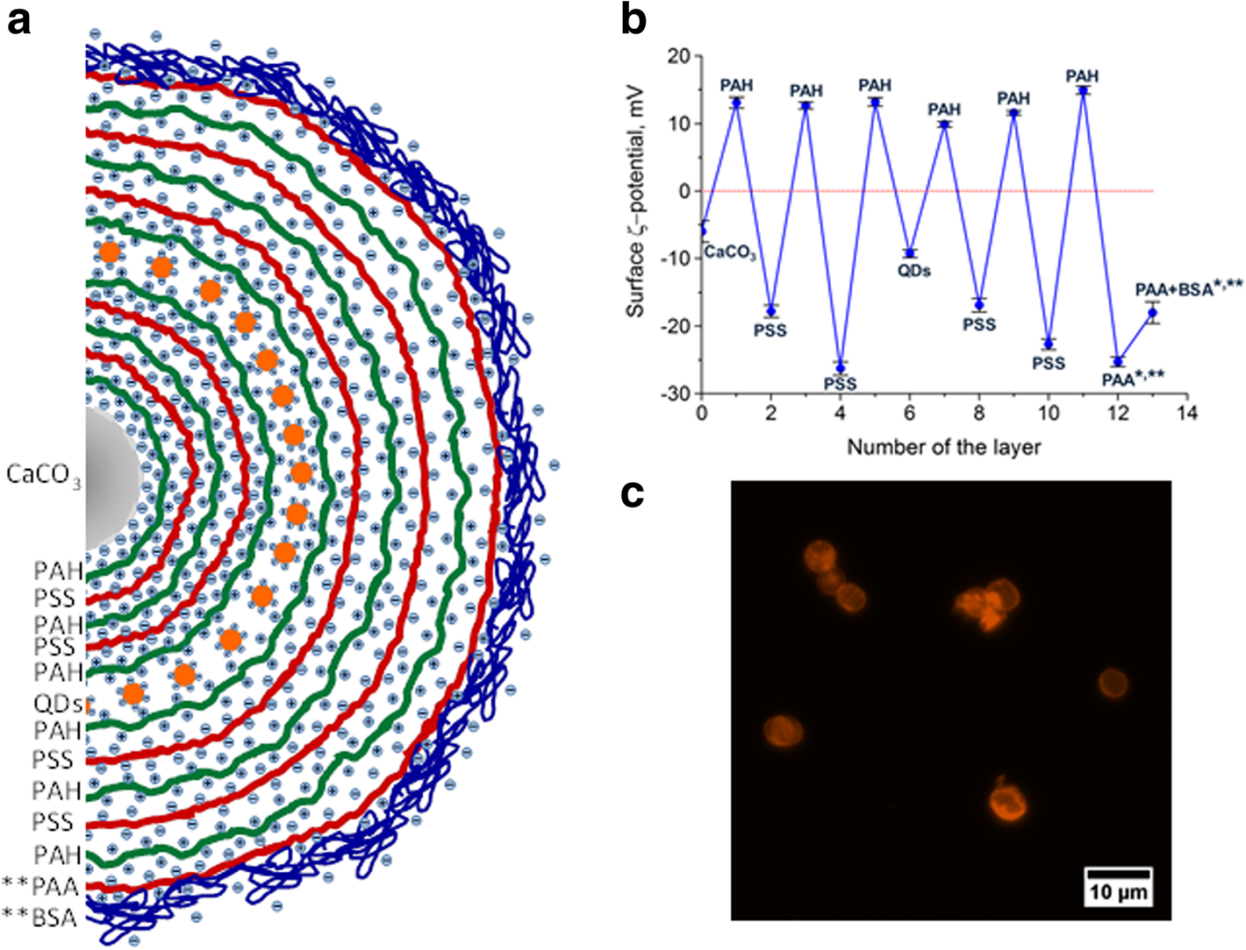
The design and structure of the polyelectrolyte microcapsules encoded with quantum dots (QDs): **a** A schematic diagram of the arrangement of the layers in the polymer microcapsule membrane. **b** Changes in the ζ-potential of the surface of the calcium carbonate microparticles during layering of polymeric electrolytes and QD encoding. **c** A fluorescent microphotograph of the polyelectrolyte microcapsules encoded with CdSe/ZnS QDs solubilized with HS-PEG_12_-COOH. * The ζ-potentials of the microcapsule surface after the core was removed; ** an additional stage in the fabrication of QD-encoded polyelectrolyte microcapsules with a BSA-modified surface

We used the layer-by-layer deposition technique for obtaining bioimaging agents in the form of QD-encoded fluorescent microparticles because the suggested method did not require organic solvents, allowed the use of biocompatible polymers [23, 24], and ensured efficient immobilization of QDs within the polymer shell [21]. The fabrication of the fluorescent polyelectrolyte microcapsules optically encoded with water-soluble, surface-modified QDs consists in application of five oppositely charged polyelectrolyte layers onto the surface of calcium carbonate microparticles serving as matrices as the first step, followed by encoding the polyelectrolyte shell with negatively charged QDs, coating the QD layer with protective layers of oppositely charged polyelectrolytes, dissolution of the core matrix of the microparticle, and, finally, modification of the microcapsule surface with BSA (Fig. 1). The negative surface charge of the calcium carbonate microparticles ensures the adsorption of PAH due to electrostatic interaction of the polycation with the microparticle surface. The positive ζ-potential of the surface resulting from PAH adsorption onto the particles allows the subsequent application of the PSS polyanion, as well as QDs modified with HS-PEG_12_-COOH, which also have a negative surface charge due to the carboxyl group and, hence, can be adsorbed onto the PAH polycation layer. Embedding of the QDs in the polymeric polyelectrolyte membrane is performed by applying additional covering polyelectrolyte layers (at least four to six of them), as shown in Fig. 1a, b.

Hollow microcapsules containing immobilized QDs in their polymer membrane are obtained by dissolving the calcium carbonate microparticles with 0.2 M EDTA (pH 6.5) and formation of the water-soluble complex of calcium salt of ethylenediaminetetraacetic acid, whose diffusion through the polymer membrane results in formation of cavities inside the microcapsules. For obtaining QD-encoded polyelectrolyte microcapsules with a BSA-modified surface, the PAA polyanion is layered onto the 11th layer of the PAH polycation. PAA is used because of its value of the dissociation constant upon interaction with the microcapsule surface. pKa value of PAA (pKa ≈ 4.7) is lower and, hence, more acidic compared to PSS (pKa ≈ 7.5) [25, 26], which results in a higher ζ-potential of the microcapsule surface. The charge of the PAA-modified surface facilitates BSA passive adsorption due to electrostatic interaction between BSA and PAA. However, assembly between PAA and BSA results in microcapsule negative surface charge decrease (Fig. 1b). After BSA deposition onto the microcapsule surface, the shielding of negatively charged PAA layer by electrostatically positive amino groups of BSA occurs, hence, the ζ-potential of the BSA-coated QD-encoded microcapsules is likely primary determined by electrostatic behavior of BSA as an outer microcapsule coating [26].

The water-soluble PEGylated QDs are characterized by a narrow size distribution, the absence of aggregations in the dispersion, and a high colloidal stability. This is likely to ensure homogeneous adsorption of QDs on the microbead surface and facilitate effective encoding and, hence, obtaining bright fluorescent microcapsules (Fig. 1c).

The use of proteins, such as BSA, for surface modification makes the polymer microcapsules more biocompatible and more resistant to adhesion with one another. This also ensures temporary passivation of the microcapsule surface, which is important for the subsequent study of the microcapsules formed by PAH–PSS or PAH–PAA interpolymer complexes in terms of in vitro interaction with cells and in vivo behavior [27–29].

**Fig. 2:**
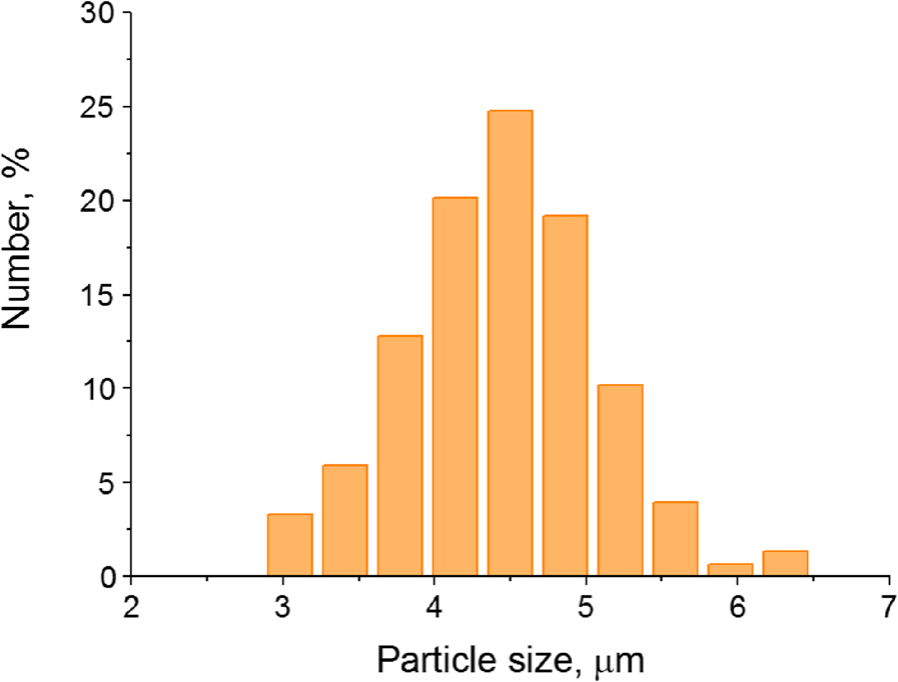
Size distribution of the polyelectrolyte microcapsules optically encoded with quantum dots, where the number of the analyzed microcapsules was 600

The obtained QD-encoded microcapsules are spherical (Fig. 1c) and are characterized by a narrow size distribution (Fig. 2) with a mean size of 4.45 ± 0.65 μm. This size is comparable with that of red blood cells, being even smaller than it [30]. In addition, as shown earlier, the polymer membrane of the microcapsules is a flexible structure prone to deformation. Being injected intravenously, microcapsules of this size cannot penetrate across blood–tissue barriers, which allows the transport and distribution of optically encoded microcapsules in the body to be traced [31, 32]. However, in localizations with an enhanced permeability of the blood vessel wall, e.g., in inflammation and tumor-growth areas, microcapsules can penetrate into the extravascular space, which is expected to ensure the imaging and monitoring of targeted delivery [33–36].

### The Fluorescence and Structure Characteristics of the QD-Encoded Polyelectrolyte Microcapsules

**Fig. 3:**
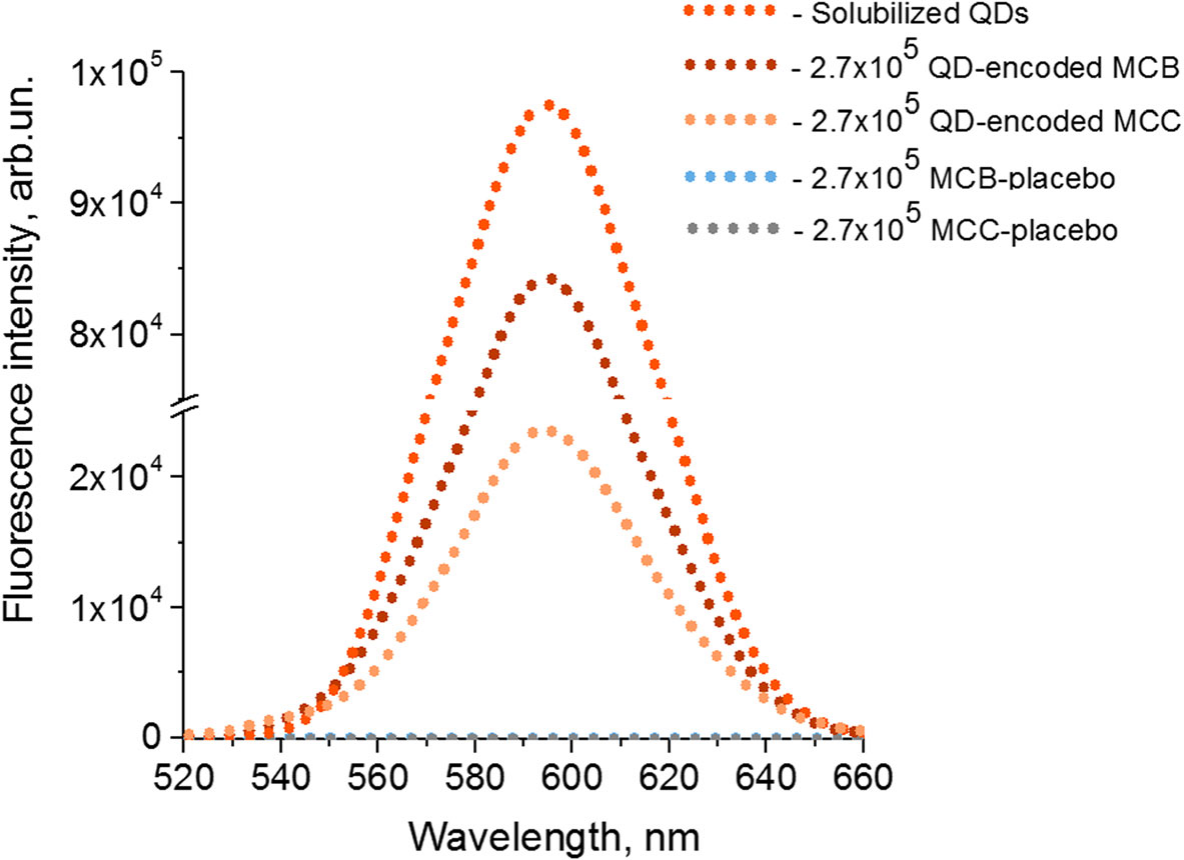
The effect of quantum dot (QD) incorporation into the polymer membrane of the microbeads (MCBs) and microcapsules (MCCs) on their fluorescence characteristics: The fluorescence spectrum of a QD solution containing 2.241 mg of QDs is shown; this corresponds to the amount of QDs used for the optical encoding of the MCBs

The fabricated microcapsules are characterized by a fluorescence peak at a wavelength of 590 nm, which corresponds to that of the original water-soluble QDs used for the optical encoding of the microcapsules. This indicates that the polymeric polyelectrolytes constituting the microcapsule membrane do not affect the fluorescent properties, particularly fluorescent maximum, of the QDs in the microbeads and the microcapsules prepared from them (Fig. 3).

**Fig. 4:**
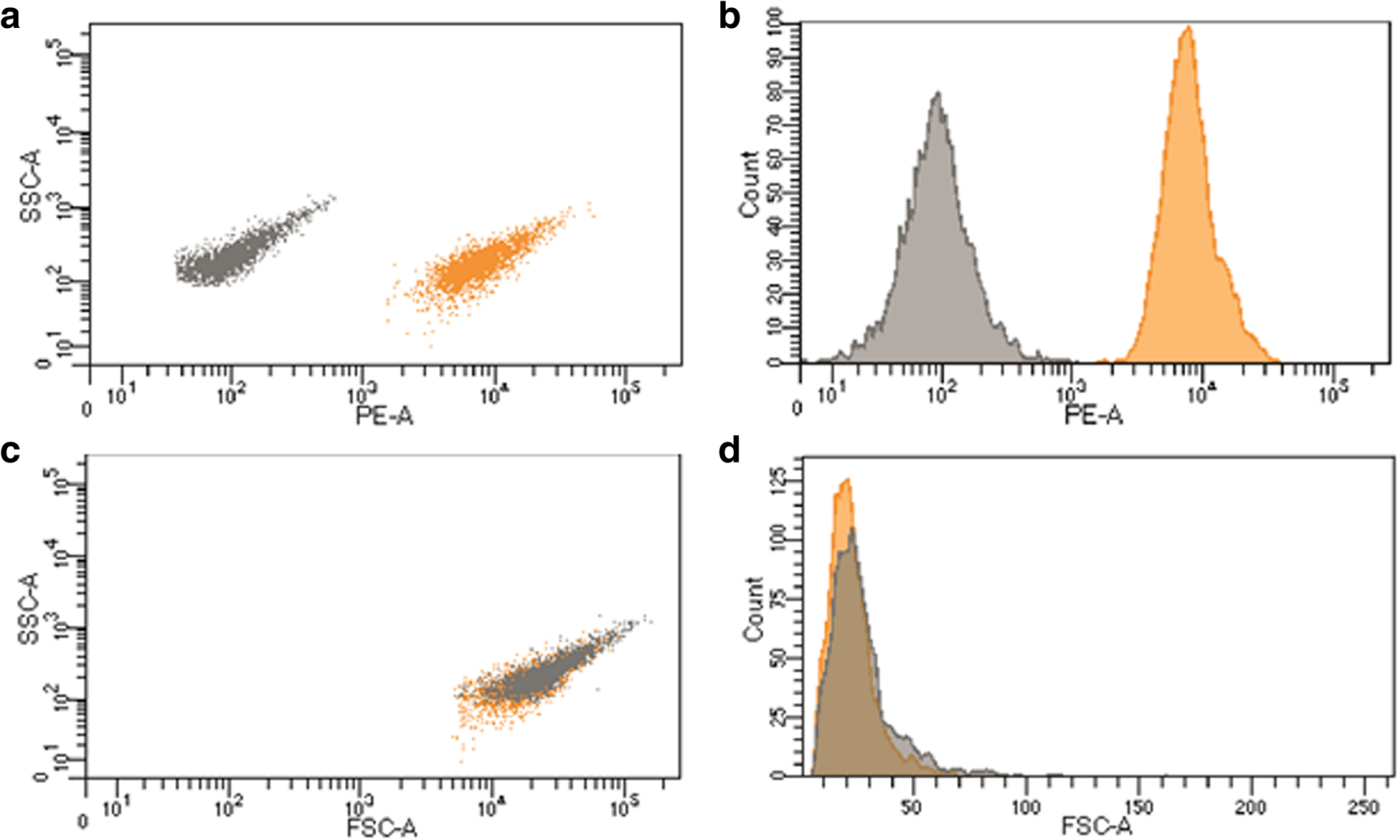
Detectability of QD-encoded polyelectrolyte microcapsules by flow cytometry: **a** microcapsule dot-plot profile in SSC-PE channels; **b** microcapsule distribution histogram in PE channel; **c** microcapsule dot-plot profile in SSC-FSC channels; **d** microcapsule distribution histogram in FSC channel. QD-free microcapsules (placebo) were used as a control and are shown in gray, whereas those encoded with CdSe/ZnS quantum dots (fluorescence emission maximum at 590 nm) are shown in orange. The number of analyzed events was equal to 2500. The dot-plots and histogram axes are shown as SSC-A, FSC-A, PE-A, where A means the data are represented by signal area

Figure 4 shows the distribution histograms for the intensity of signals from the populations of QD-encoded microcapsules, as well as placebo microcapsules, in the PE (575/25 nm) fluorescence channel and FSC-A and SSC-A (488/10 nm) channels of the flow cytometer. The data indicate effective differentiation between the placebo and optically encoded microcapsules in the PE (575/25 nm) channel (Fig. 4a, b). The intensity of the fluorescence signal from the microcapsules in the PE (575/25 nm) channel is ~ 10^4^, which demonstrates a high fluorescence capacity of the QD-encoded microcapsules. In the FSC-A and SSC-A (488/10 nm) channels, the distributions for the two microcapsule populations overlap, which indicates similar relative sizes and granularity parameters of the placebo and encoded microcapsules (Fig. 4c, d) and, hence, homogeneity of the populations. Apparently, this is due to the fact the microcapsule membranes consist of equal numbers of polymer layers, the membrane of the encoded microcapsules containing only one layer of QDs. Thus, the obtained microcapsules are characterized by a homogeneous size distribution and optimal fluorescence characteristics ensuring their detection in the corresponding channels of a flow cytometer.

**Fig. 5:**
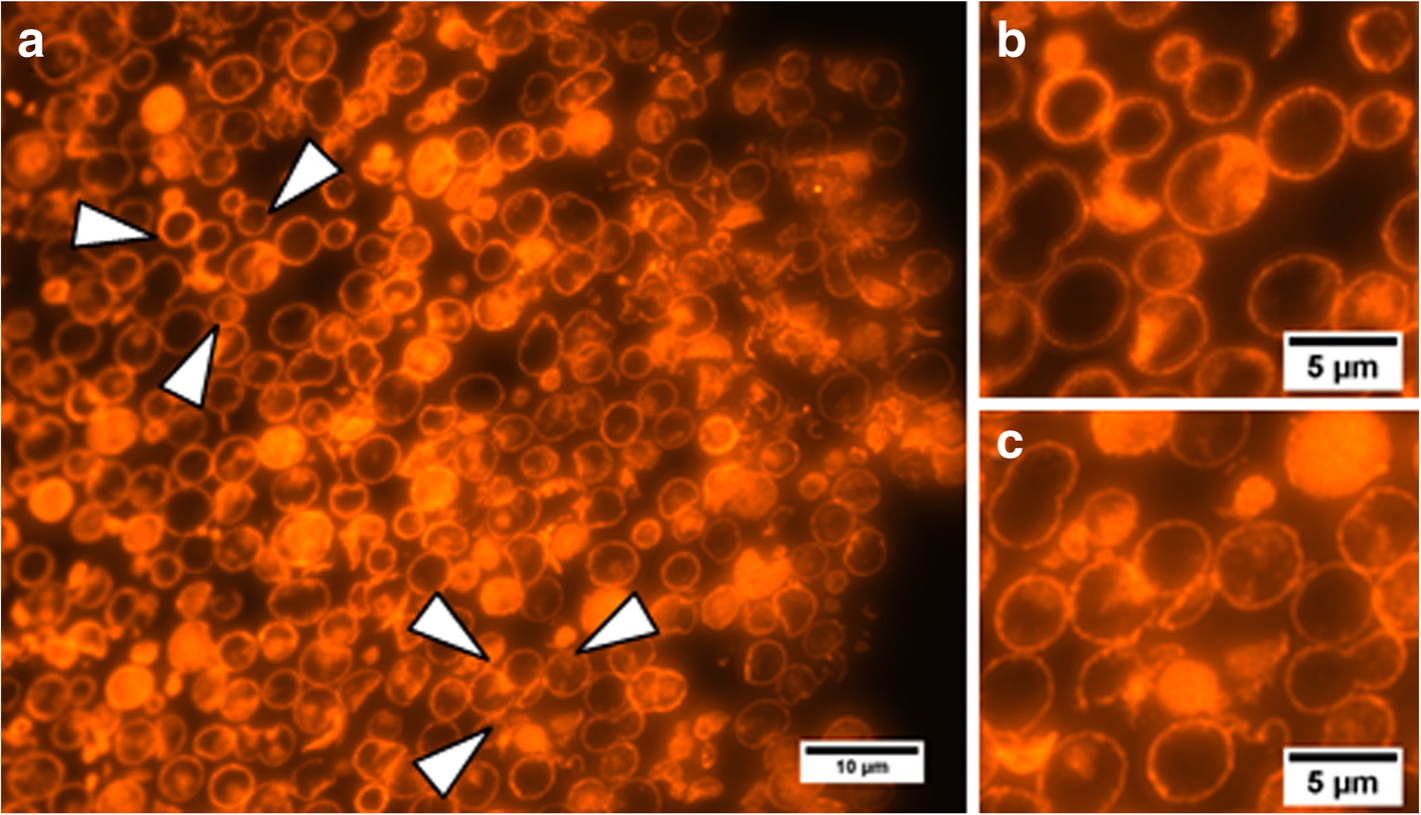
Microphotographs of sections of the polyelectrolyte microcapsules encoded with quantum dots. The arrows in (**a**) indicate the areas shown in (**b**, **c**) at a higher magnification

The microphotographs of sections of the QD-encoded polyelectrolyte microcapsules shown in Fig. 5 demonstrate that the microcapsules are hollow, the detected bright fluorescent signal being emitted by the polymer membranes containing QDs. These data confirm the efficiency of the procedure used for dissolving the core and demonstrate a bright fluorescence signal of the fabricated microcapsules, which can be detected using the corresponding filters, Texas Red and PE in the cases of fluorescence microscopy and flow cytometry, respectively. The prepared polyelectrolyte microcapsules containing QDs in its polymer shell possess higher fluorescent properties comparing to polyelectrolyte microcapsules labeled with conventional dyes such as fluorescein isothiocyanate (FITC) or aminofluorescein [14, 37]. Otherwise, the microcapsules encoded with QDs using the layer-by-layer approach have the brightness comparable or even inferior than that for the microbeads encoded with organic dyes using swelling technique. The brightness is determined by the product of extinction coefficient and the quantum yield. The quantum yields of water-soluble QDs at room temperature are around 40%, what is comparable with that for organic dyes [22, 38, 39], whereas QDs extinctions are nearly 100-times bigger than that for organic dyes. Otherwise, due to the big size of QDs, their quantities within the microcapsule shell may not be made comparable with that for organic dyes molecules. So, the quantities of encoding organic dye molecules may be made much superior than that for QDs thus ensuring comparable brightness. From the other side, encoding of the microcapsules with the QDs provides such important comparative advantage as the complete absence of the photobleaching. Additionally, the QDs of different colors (sizes) may be excited with the same wavelength excitation. So, the use of the QDs of different colors for microcapsules encoding may provide practically unlimited number of spectrally resolved optical codes [21].

## Conclusions

Developed technique for obtaining QD-encoded polyelectrolyte microcapsules ensures effective optical encoding. The fabricated polymer microcapsules are characterized by an optimal size distribution and high fluorescence intensity to be used for their efficient detection by commercial flow cytometers and confocal microscopes. Therefore, the designed microcapsules are potential fluorescent agents for in vitro and in vivo bioimaging. Further development of the versatile microcapsule-based platform would be aimed to proposition of novel bioimaging and theranostic tools based on fluorescent QD-encoded microparticles which are responsive to different external physical or chemical stimuli along with photoexcitation.

## Abbreviations

BSA: Bovine serum albumin
EDTA: Disodium ethylenediaminetetraacetate
PAA: Polyacrylic acid
PAH: Polycation poly(allylamine hydrochloride)
PEG: Polyethylene glycol
PSS: Polyanion poly(sodium 4-styrenesulfonate)
QD: Quantum dot
TOPO: Trioctylphosphine oxide
